# Quercetin Ameliorates Acute Lethal Sepsis in Mice by Inhibiting Caspase-11 Noncanonical Inflammasome in Macrophages

**DOI:** 10.3390/molecules29245900

**Published:** 2024-12-13

**Authors:** Eojin Kim, Deok-Hyeong Choi, Young-Su Yi

**Affiliations:** Department of Life Sciences, Kyonggi University, Suwon 16227, Republic of Korea; eojin-kim@naver.com (E.K.); korean236@naver.com (D.-H.C.)

**Keywords:** quercetin, caspase-11, noncanonical inflammasome, anti-inflammatory, acute lethal sepsis, macrophage

## Abstract

Quercetin is a natural polyphenolic flavonoid widely found in plants, fruits, and vegetables, and has been reported to play pharmacological roles in numerous pathogenic conditions. The anti-inflammatory effects of quercetin in various inflammatory conditions and diseases have been well-documented. However, its regulatory role in noncanonical inflammasome activation has not yet been demonstrated. This study investigated the anti-inflammatory effects of quercetin in caspase-11 noncanonical inflammasome-activated inflammatory responses in macrophages and a mouse model of acute lethal sepsis. Quercetin protected J774A.1 macrophages from lipopolysaccharide (LPS)-induced cell death and caspase-11 noncanonical inflammasome-induced pyroptosis. It significantly decreased the production and mRNA expression of pro-inflammatory cytokines, such as interleukin (IL)-1β, IL-18, and IL-6, but not tumor necrosis factor (TNF)-α, and inflammatory molecules, such as nitric oxide (NO) and inducible NO synthase in caspase-11 noncanonical inflammasome-activated J774A.1 cells. Mechanistically, quercetin strongly suppressed the autoproteolysis and secretion of caspase-11 and the proteolysis of gasdermin D in caspase-11 noncanonical inflammasome-activated J774A.1 cells. However, quercetin did not inhibit the direct binding of caspase-11 to LPS. In vivo, the study revealed that quercetin increased the survival rate of mice with acute lethal sepsis and decreased serum levels of pro-inflammatory cytokines without causing significant toxicity. In conclusion, this study highlights quercetin-mediated anti-inflammatory action in inflammatory responses and acute lethal sepsis through a novel mechanism that targets the caspase-11 noncanonical inflammasome in macrophages, suggesting quercetin as a promising anti-inflammatory agent in natural medicine.

## 1. Introduction

Inflammation is a natural innate immune response that occurs in two steps: priming and triggering [1,2]. During the priming step, the immune system prepares for an inflammatory response by upregulating the gene expression of inflammatory molecules, such as pro-inflammatory cytokines and inflammatory enzymes. The triggering step subsequently activates and boosts this inflammatory response through the formation and activation of inflammasomes—cytoplasmic multiprotein complexes of pattern-recognition receptors (PRRs) and inflammatory molecules such as inflammatory caspases and apoptosis-associated speck-like protein containing a CARD (ASC) [3]. Intracellular PRRs detect various pathogen-associated molecular patterns (PAMPs) and danger-associated molecular patterns (DAMPs), followed by the formation and activation of inflammasomes [4]. Previously identified canonical inflammasomes include NLR family inflammasomes, such as NLRP1, NLRP3, NLRC4, NLRP6, and NLRP9, as well as non-NLR family inflammasomes, such as absent in melanoma 2 and pyrin inflammasomes [4]. Recently identified noncanonical inflammasomes include mouse caspase-11 and its human counterparts, caspase-4, and caspase-5 [5,6,7]. Although PAMPs and DAMPs differ between canonical and noncanonical inflammasomes, inflammasomes share downstream inflammatory signaling pathways. Activation of canonical and noncanonical inflammasomes promotes the site-specific cleavage of gasdermin D (GSDMD) at aspartic acid residue 276, leading to the recruitment of cleaved amino-terminal fragments of GSDMD (N-GSDMD) to cell membranes, leading to GSDMD pore formation and GSDMD pore-mediated pyroptosis, an inflammatory form of cell death [8,9]. Simultaneously, the activation of noncanonical inflammasomes induces autoproteolytic cleavage of caspase-4/11, and the activated caspase-4/11 noncanonical and canonical inflammasomes promotes the proteolytic activation of caspase-1, leading to proteolytic maturation and secretion of pro-inflammatory cytokines, interleukin (IL)-1β, and IL-18 through GSDMD pores [8,9]. Since the discovery of canonical inflammasomes, many studies have demonstrated their critical roles in the pathogenesis of various human diseases by modulating inflammatory responses [10,11,12,13,14,15]. Recent studies have also revealed that noncanonical inflammasomes play key roles in inflammatory responses and are implicated in various human diseases, including acute lethal sepsis, rheumatic diseases, gastrointestinal diseases, and inflammatory liver diseases [16,17,18,19,20,21,22,23,24,25]. However, the roles of noncanonical inflammasomes in many human diseases, their underlying mechanisms, and their validation as therapeutic targets remain largely unknown.

Quercetin [2-(3,4-Dihydroxyphenyl)-3,5,7-trihydroxy-4H-1-benzopyran-4-one], a plant flavonol from the flavonoid group of polyphenols, is widely found in many fruits, vegetables, leaves, grains, seeds, and tea. It exhibits various pharmacological properties, including antioxidant, antimicrobial, antiviral, anticancer, antidiabetic, antiarthritic, cardiovascular protective, gastroprotective, hepatoprotective, and neuroprotective effects [26,27,28]. The anti-inflammatory properties of quercetin have also been demonstrated [29,30,31,32]. However, research on the anti-inflammatory roles of quercetin has mostly focused on the priming step of inflammatory responses and canonical inflammasomes [29,30,31,32]. To date, no studies have investigated the role of quercetin in noncanonical inflammasome-activated inflammatory responses and diseases. This study demonstrated the in vitro and in vivo anti-inflammatory roles of quercetin in caspase-11 noncanonical inflammasome-activated macrophages and in a mouse model of acute lethal sepsis induced by noncanonical inflammasome activation.

## 2. Results

### 2.1. Quercetin Prevented Pyroptosis Induced by Caspase-11 Noncanonical Inflammasome Activation in Macrophages

**Figure 1:**
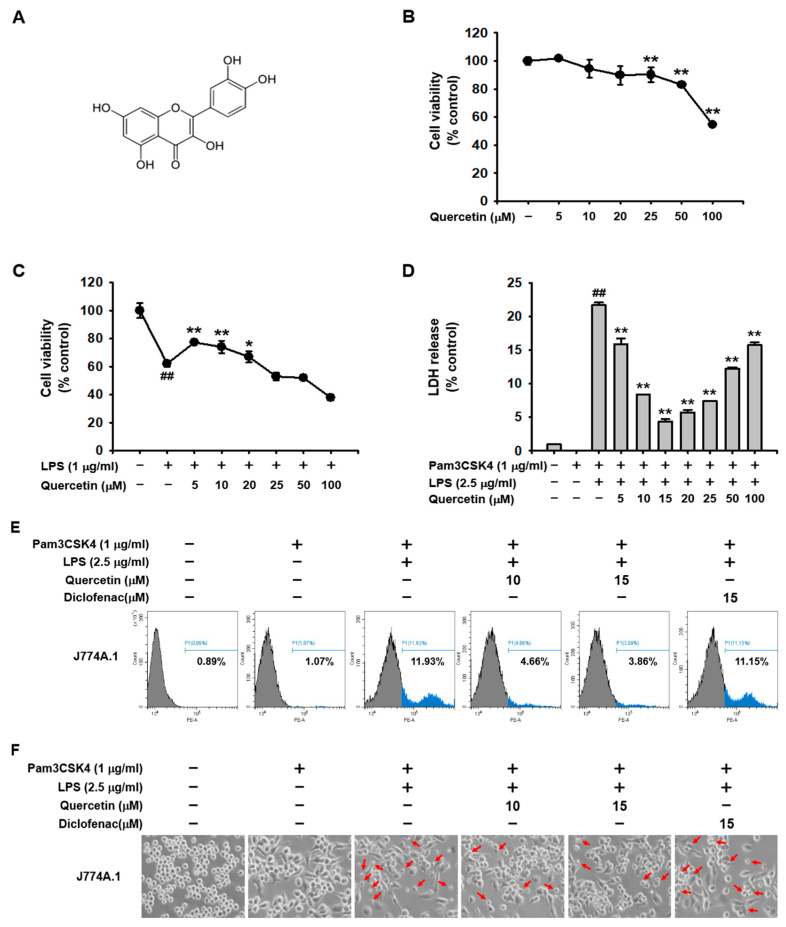

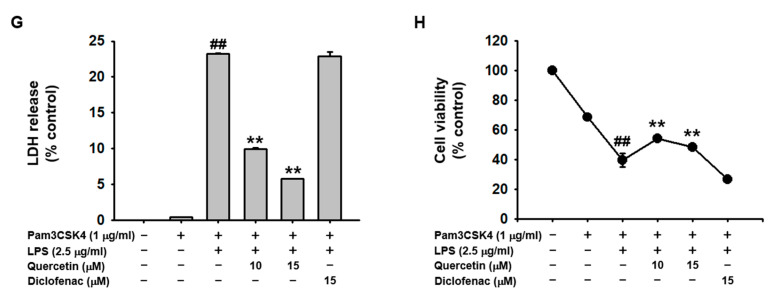
Quercetin prevents pyroptosis induced by caspase-11 noncanonical inflammasome activation in macrophages. (**A**) Chemical structure of quercetin. (**B**) J774A.1 cells were treated with quercetin (5, 10, 20, 25, 50, and 100 μM) for 24 h, and cell viability was determined using an MTT assay. (**C**) J774A.1 cells pretreated with quercetin (5, 10, 20, 25, 50, and 100 μM) for 1 h were then treated with LPS (1 μg/mL) for 24 h, and cell viability was determined using an MTT assay. (**D**) J774A.1 cells pretreated with quercetin (5, 10, 15, 20, 25, 50, and 100 μM) for 1 h were treated with Pam3CSK4 (1 μg/mL) for 4 h and subsequently transfected with LPS (2.5 μg/mL) for 20 h. LDH levels released from the J774A.1 cells were determined. J774A.1 cells pretreated with either quercetin (10 and 15 μM) or diclofenac (15 μM) for 1 h were treated with Pam3CSK4 (1 μg/mL) for 4 h and transfected with LPS (2.5 μg/mL) for 20 h. (**E**) The cells were stained with PI, and the PI-stained cells were analyzed by flow cytometry. Numbers indicate % of pyroptotic cell death. (**F**) Cell morphology was observed and photographed under a light microscope. Red arrows indicate pyroptotic cells. (**G**) LDH levels released from J774A.1 cells were measured. (**H**) Cell viability was determined using an MTT assay. ** *p* < 0.01 compared to a (−) control (**B**). ^##^ *p* < 0.01 compared to a (−) control, * *p* < 0.05 and ** *p* < 0.01 compared to an LPS-treated control (**C**). ^##^ *p* < 0.01 compared to Pam3CSK4-treated controls, and ** *p* < 0.01 compared to LPS-transfected controls (**D**,**F**,**G**). Arrows indicate pyroptotic death of J774A.1 cells.

To examine the cytotoxicity of quercetin in macrophages, J774A.1 cells were treated with various doses of quercetin for 24 h, followed by a cell viability assay. Quercetin showed no cytotoxicity up to 20 μM, mild cytotoxicity at 25 and 50 μM, and severe cytotoxicity at 100 μM in J774A.1 cells (Figure 1B). The protective effects of quercetin against LPS-mediated cytotoxicity in macrophages were examined. The results showed that Quercetin protected J774A.1 cells from LPS-mediated cytotoxicity at doses of 5, 10, and 20 μM but not at 25 μM (Figure 1C). The inhibitory effect of quercetin on caspase-11 noncanonical inflammasome-activated pyroptosis was then examined. Quercetin significantly inhibited the LDH release in J774A.1 cells in a dose-dependent manner up to 15 μM, but its inhibitory effect decreased at doses from 20 μM to 100 μM (Figure 1D). Higher LDH release from 20 μM might be caused by the decreasing cell viability when using higher doses of quercetin. Based on these results, doses of 10 and 15 mM were selected for further experiments. The inhibitory effect of quercetin on pyroptosis in macrophages was further analyzed with diclofenac, an FDA-approved non-steroidal anti-inflammatory drug (NSAID), to compare anti-inflammatory activity since diclofenac is a widely known anti-inflammatory drug that is currently in use. Quercetin effectively prevented J774A.1 cell death induced by caspase-11 noncanonical inflammasome activation (Figure 1E,F) and suppressed pyroptosis-mediated release of LDH (Figure 1G). Furthermore, quercetin increased J774A.1 cell viability decreased by caspase-11 noncanonical inflammasome activation (Figure 1H). However, diclofenac showed neither inhibitory (Figure 1E–G) nor protective effects (Figure 1H) against pyroptosis in these cells. These results suggest that quercetin plays a protective role against caspase-11 noncanonical inflammasome-activated pyroptosis in macrophages.

### 2.2. Quercetin Suppressed the Production of Inflammatory Mediators Induced by Caspase-11 Noncanonical Inflammasome Activation in Macrophages

**Figure 2:**
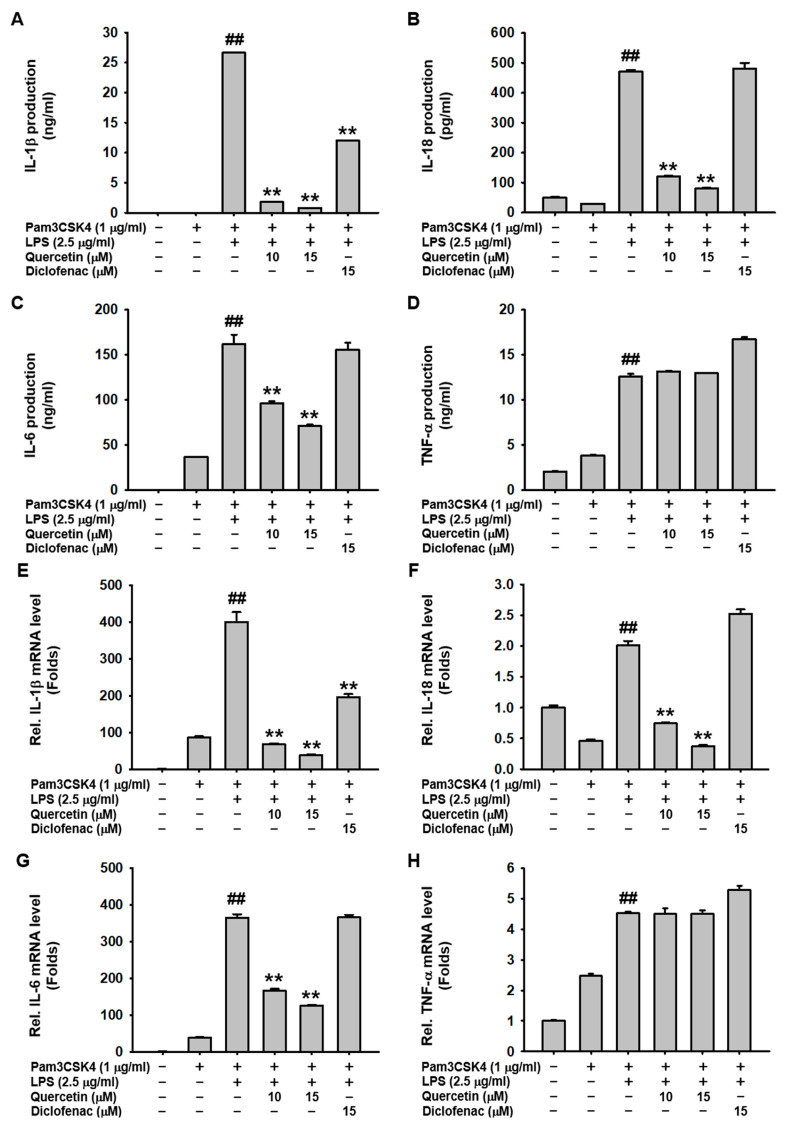

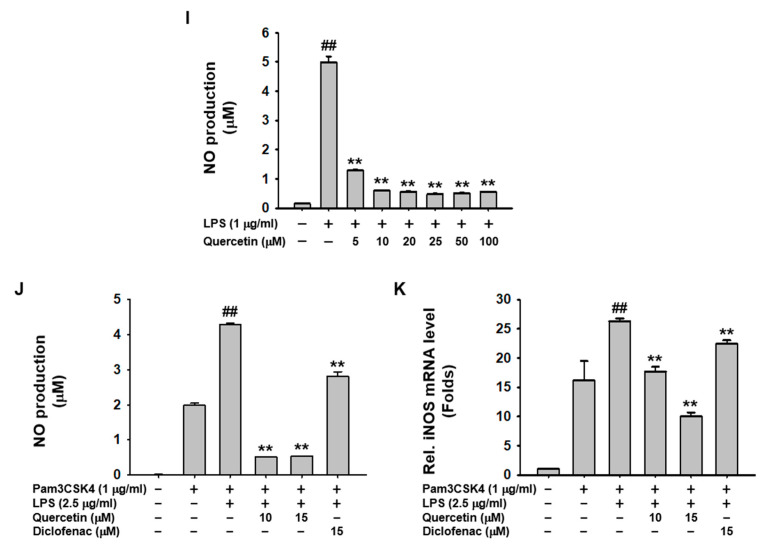
Quercetin suppresses the production of inflammatory mediators induced by caspase-11 noncanonical inflammasome activation in macrophages. J774A.1 cells pretreated with either quercetin (10 and 15 μM) or diclofenac (15 μM) for 1 h were treated with Pam3CSK4 (1 μg/mL) for 4 h and transfected with LPS (2.5 μg/mL) for 20 h. (**A**) IL-1β, (**B**) IL-18, (**C**) IL-6, and (**D**) TNF-α released in the cell culture media were quantified by ELISA. mRNA expressions of (**E**) IL-1β, (**F**) IL-18, (**G**) IL-6, (**H**) TNF-α, and (**K**) iNOS were determined by qPCR. (**I**) J774A.1 cells pretreated with quercetin (5, 10, 20, 25, 50, and 100 μM) for 1 h were treated with LPS (1 μg/mL) for 24 h, and NO levels in the cell culture media were determined by a Griess assay. (**J**) J774A.1 cells pretreated with either quercetin (10 and 15 μM) or diclofenac (15 μM) for 1 h were treated with Pam3CSK4 (1 μg/mL) for 4 h and transfected with LPS (2.5 μg/mL) for 20 h, and NO levels in the cell culture media were determined by a Griess assay. ^##^ *p* < 0.01 compared to a (−) control, and ** *p* < 0.01 compared to an LPS-treated control (**I**). ^##^ *p* < 0.01 compared to Pam3CSK4-treated controls, and ** *p* < 0.01 compared to LPS-transfected controls (**A**–**H**,**J**,**K**).

The inhibitory effects of quercetin on the production of inflammatory molecules were examined in caspase-11 noncanonical inflammasome-activated macrophages. Quercetin significantly inhibited the secretion of pro-inflammatory cytokines, such as IL-1β, IL-18, and IL-6, but had no effect on TNF-α secretion in caspase-11 noncanonical inflammasome-activated J774A.1 cells (Figure 2A–D). Consistent with these results, quercetin decreased the mRNA expression of IL-1β, IL-18, and IL-6, but did not decrease the mRNA expression of TNF-α (Figure 2E–H). Additionally, Quercetin inhibited NO production in both LPS-stimulated and caspase-11 noncanonical inflammasome-activated J774A.1 cells (Figure 2I,J). This inhibition was further supported by a decrease in iNOS mRNA expression in the activated macrophages (Figure 2K). Diclofenac also inhibited NO production and mRNA expression of iNOS in caspase-11 noncanonical inflammasome-activated J774A.1 cells, but its inhibitory effect was significantly less potent than that of quercetin (Figure 2J,K). These results suggest that quercetin inhibits the production and mRNA expression of pro-inflammatory cytokines and other inflammatory molecules in caspase-11 noncanonical inflammasome-activated macrophages.

### 2.3. Quercetin-Mediated Inhibitory Mechanisms of Caspase-11 Noncanonical Inflammasome in Macrophages

**Figure 3:**
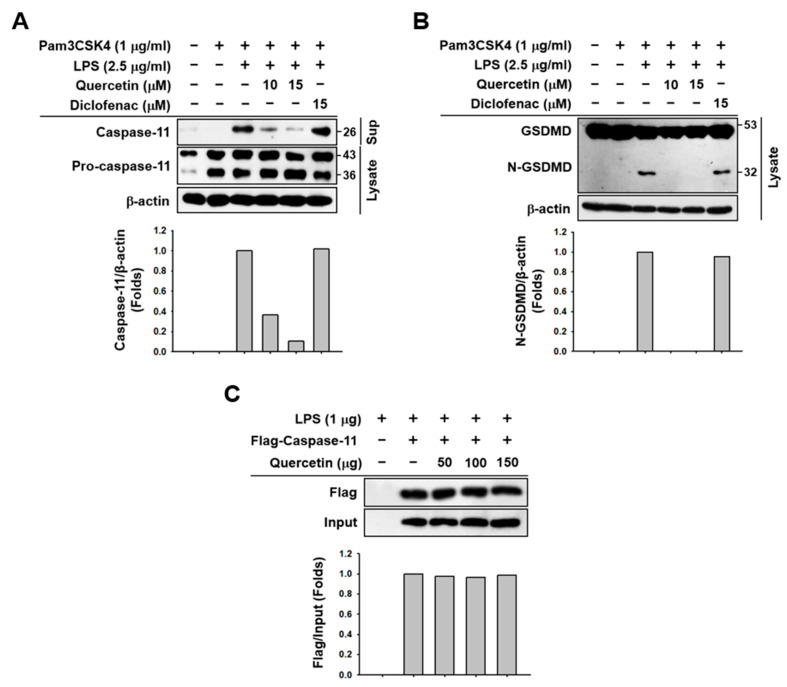
Quercetin-mediated inhibitory mechanisms of caspase-11 noncanonical inflammasome in macrophages. J774A.1 cells pretreated with either quercetin (10 and 15 μM) or diclofenac (15 μM) for 1 h were treated with Pam3CSK4 (1 μg/mL) for 4 h and transfected with LPS (2.5 μg/mL) for 20 h. (**A**) Uncleaved and cleaved forms of caspase-11 in the whole cell lysates and cell culture media detected by Western blot analysis. (**B**) Uncleaved and cleaved forms of GSDMD in the whole cell lysates detected by Western blot analysis. (**C**) Direct interaction between Flag-caspase-11 expressed in HEK293 cells and biotin conjugated LPS (1 μg) in the absence or presence of quercetin (50, 100, and 150 μg) was detected by Western blot analysis. The bands were quantified and plotted using an ImageJ software (version 1.54k).

The molecular mechanisms by which quercetin inhibits pyroptosis and the production of inflammatory molecules were investigated in macrophages. Quercetin was found to dose-dependently inhibit the proteolytic cleavage of pro-caspase-11 and the secretion of cleaved caspase-11 in J774A.1 cells (Figure 3A). Additionally, quercetin strongly inhibited the proteolytic cleavage of GSDMD and the formation of N-GSDMD in these cells (Figure 3B). Notably, diclofenac showed no inhibitory effect on the proteolytic cleavage of either pro-caspase-11 or GSDMD, nor did it affect the secretion of caspase-11 in J774A.1 cells (Figure 3A,B). However, quercetin did not inhibit the direct binding between caspase-11 and LPS (Figure 3C). These results suggest that quercetin plays an anti-inflammatory role by inhibiting pyroptosis and the activation and release of inflammatory molecules by suppressing the proteolytic activation of caspase-11 and GSDMD in macrophages.

### 2.4. Quercetin Ameliorated LPS-Induced Acute Lethal Sepsis in Mice

**Figure 4:**
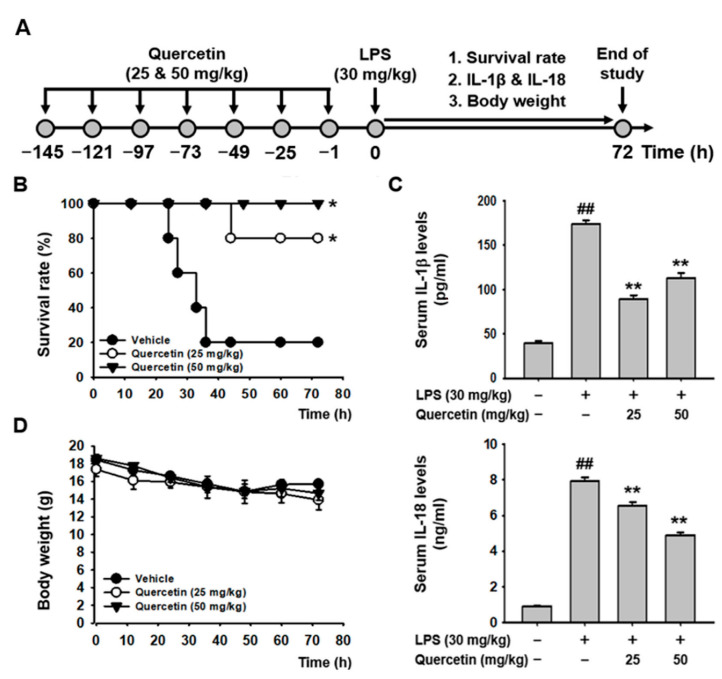
Quercetin ameliorates LPS-induced acute lethal sepsis in mice. (**A**) Experimental design and schedule. C57BL/6 mice were intraperitoneally injected with quercetin (25 and 50 mg/kg) every 24 h for 7 days, followed by an intraperitoneal injection of LPS (30 mg/kg). (**B**) The survival rates, and (**D**) the body weights measured for 72 h. (**C**) Serum levels of IL-1β and IL-18 measured at 72 h in the mice. * *p* < 0.05 compared to vehicle-injected controls (**B**). ^##^ *p* < 0.01 compared to vehicle-injected controls, and ** *p* < 0.01 compared to LPS-injected controls (**C**).

The in vivo anti-inflammatory effects of quercetin were examined using a mouse model of LPS-induced acute lethal sepsis. Mice were intraperitoneally injected with quercetin (25 and 50 mg/kg) every 24 h for a total of seven doses. One hour after the final injection, acute lethal sepsis was induced by intraperitoneal injection of LPS (30 mg/kg). The survival rate and body weight of the mice were monitored for 72 h, and blood was collected to examine the serum levels of pro-inflammatory cytokines after sacrificing the mice at the end of the observation period (Figure 4A). Quercetin significantly increased the survival rates of the diseased mice at both doses, with a 100% survival rate observed at 50 mg/kg (Figure 4B). Correspondingly, quercetin significantly reduced serum levels of IL-1β and IL-18 in the mice with acute lethal sepsis (Figure 4C). Body weight, however, did not differ significantly between the vehicle-injected control group and quercetin-injected groups, nor between the two doses of quercetin in the acute lethal septic mice (Figure 4D). These results suggest that quercetin plays an in vivo anti-inflammatory role, ameliorating acute lethal sepsis by increasing survival rates and inhibiting the production of pro-inflammatory cytokines in mice with acute lethal sepsis without significant toxicity at the tested doses. The *p* values for survival rate comparisons between groups are listed in Table 1.

## 3. Materials and Methods

### 3.1. Cell Culture

The mouse macrophage cell line J774A.1 (American Type Culture Collection, Manassas, VA, USA) was cultured in Roswell Park Memorial Institute 1640 medium (Thermo Fisher Scientific, Waltham, MA, USA) with 10% heat-inactivated fetal bovine serum (FBS) and an antibiotic solution containing 100 units penicillin, 0.29 mg/mL streptomycin, and 0.1 mM L-glutamine (Thermo Fisher Scientific, Waltham, MA, USA). HEK293 cells (American Type Culture Collection, Manassas, VA, USA) were cultured in Dulbecco’s modified Eagle’s medium (Thermo Fisher Scientific, Waltham, MA, USA) supplemented with 10% FBS and an antibiotic solution (Thermo Fisher Scientific, Waltham, MA, USA). The cells were cultured in a 37 °C humidified incubator supplemented with 5% CO_2_.

### 3.2. Cell Viability Assay

J774A.1 cells treated with the indicated doses of quercetin (Figure 1A; Alfa Aesar, Ward Hill, MA, USA) for 24 h. The cells were treated with 1 μg/mL lipopolysaccharide (LPS; Thermo Fisher Scientific, Waltham, MA, USA) and the indicated doses of quercetin for 24 h. The cells pretreated with either the indicated doses of quercetin or 15 μM diclofenac (Alfa Aesar, Ward Hill, MA, USA) for 1 h were treated with 1 μg/mL Pam3CSK4 (InvivoGen, San Diego, CA, USA) for 4 h, followed by transfection with 2.5 μg/mL LPS using Fugene HD (Promega, Madison, WI, USA) for 20 h. The viability of cells was determined using the 3-(4,5-dimethylthiazole-2-yl)-2,5-diphenyl tetrazolium bromide (MTT) assay (HanLAB, Cheongju, Republic of Korea), as previously described [33].

### 3.3. Lactate Dehydrogenase (LDH) Assay

J774A.1 cells pretreated with either the indicated doses of quercetin or 15 μM diclofenac for 1 h were treated with 1 μg/mL Pam3CSK4 for 4 h, followed by transfection with 2.5 μg/mL LPS using Fugene HD for 20 h. The amount of LDH in the culture media was measured using the Quanti-LDH PLUS Cytotoxicity Assay Kit (BIOMAX, Guri, Republic of Korea), and cell shapes were observed using a light microscope.

### 3.4. Flow Cytometry

J774A.1 cells pretreated with either the indicated doses of quercetin or 15 μM diclofenac for 1 h were treated with 1 μg/mL Pam3CSK4 for 4 h, followed by transfection with 2.5 μg/mL LPS using Fugene HD for 20 h. The cells were incubated with 1 μg/mL propidium iodide (Thermo Fisher Scientific, Waltham, MA, USA) in flow buffer (0.1% BSA and 0.01% sodium azide in PBS) for 10 min on ice in the dark. After five washes with flow buffer, the cells were resuspended in flow buffer, and PI-stained cells were analyzed using a CytoFLEX flow cytometer (Beckman Coulter Life Sciences, Pasadena, CA, USA).

### 3.5. Enzyme-Linked Immunosorbent Assay (ELISA)

J774A.1 cells pretreated with either the indicated doses of quercetin or 15 μM diclofenac for 1 h were treated with 1 μg/mL Pam3CSK4 for 4 h, followed by transfection with 2.5 μg/mL LPS using Fugene HD for 20 h. The amounts of IL-1β, IL-18, TNF-α, and IL-6 in the culture media and mouse blood were measured by ELISA using target-specific kits (Thermo Fisher Scientific, Waltham, MA, USA).

### 3.6. Quantitative Real-Time PCR (qPCR)

J774A.1 cells pretreated with either the indicated doses of quercetin or 15 μM diclofenac for 1 h were treated with 1 μg/mL Pam3CSK4 for 4 h, followed by transfection with 2.5 μg/mL LPS using Fugene HD for 20 h. Total RNA was extracted from the cells using an easy-BLUE reagent (iNtRON Biotechnology, Seongnam, Republic of Korea), and cDNA was immediately synthesized using M-MLV reverse transcriptase (Bioneer, Daejeon, Republic of Korea). mRNA expression levels of IL-1β, IL-18, TNF-α, and IL-6, and inducible nitric oxide synthase (iNOS) were quantified by qPCR using target-specific primers. The primer sequences are listed in Table 2.

### 3.7. Nitric Oxide (NO) Production Assay

J774A.1 cells treated with the indicated doses of quercetin for 1 h were treated with LPS (1 μg/mL) for 24 h. Additionally, J774A.1 cells pretreated with either the indicated doses of quercetin or 15 μM diclofenac for 1 h were treated with 1 μg/mL Pam3CSK4 for 4 h, followed by transfection with 2.5 μg/mL LPS for 20 h. NO levels in the culture media were determined using the Griess assay, as described previously [34].

### 3.8. Western Blot Analysis

J774A.1 cells pretreated with either the indicated doses of quercetin or 15 μM diclofenac for 1 h were treated with 1 μg/mL Pam3CSK4 for 4 h, followed by transfection with 2.5 μg/mL LPS using Fugene HD for 20 h. Total cellular proteins were extracted by lysing the cells in radioimmunoprecipitation assay buffer (150 mM NaCl, 25 mM Tris-HCl, pH 7.4, 0.5% sodium deoxycholate, 0.1% SDS, 1% Nonidet P-40, and protease inhibitor cocktail) on ice for 1 h. Total proteins secreted into the culture media were isolated by trichloroacetic acid (DUKSAN, Ansan, Republic of Korea) precipitation at 4 °C overnight, as described previously [35]. Total proteins were separated by sodium dodecyl sulfate-polyacrylamide gel electrophoresis (SDS-PAGE), and the target proteins were detected using antibodies specific to each target, followed by visualization using an ECL reagent (ELPIS-Biotech, Daejeon, Republic of Korea).

### 3.9. LPS-Caspase-11 Binding Pull-Down Assay

HEK293 cells were transfected with either pCMV-flag or pCMV-flag-caspase-11 constructs (Addgene, Watertown, MA, USA) for 48 h. The total proteins were extracted by lysing the cells with binding buffer (150 mM NaCl, 50 mM Tris-HCl, pH 7.6, 2 mM EDTA, 1% Triton X-100, and protease inhibitor cocktail) on ice for 1 h. Biotin-LPS (1 μg; Sigma-Aldrich, Burlington, MA, USA) was bound to streptavidin-agarose beads (Thermo Fisher Scientific, Waltham, MA, USA) for 1 h at 4 °C, followed by incubation with total proteins containing the indicated amounts of quercetin on ice for 1 h. The LPS-caspase-11 complexes were eluted from the agarose beads using 1× protein sample buffer and separated by SDS-PAGE. The eluted LPS-bound caspase-11 was subjected to Western blot analysis and detected using a FLAG antibody.

### 3.10. Acute Lethal Sepsis in Mice

C57BL/6 mice (female, 6 weeks old; DBL, Seoul, Republic of Korea) were intraperitoneally injected with quercetin (25 or 50 mg/kg) every 24 h for 5 days. Twenty-four hours after the final injection, the mice were intraperitoneally injected with LPS (30 mg/kg). Survival rate and body weight were monitored for 72 h (Figure 4A). After 72 h, all mice were sacrificed, and blood was immediately collected. The levels of IL-1β and IL-18 in the blood serum of normal, LPS-injected, and LPS-injected mice administered with different doses of quercetin were determined by ELISA, as described in Section 3.5 (Figure 4A). The study protocol was approved by the Kyonggi University Animal Care and Use Committee (approval Number: 2024-001).

### 3.11. Statistical Analysis

All data obtained from at least three independent experiments were presented as mean ± standard deviation. Statistical comparisons were performed using the ANOVA and post-hoc tests to assess the differences in the means between multiple groups. A *p*-value of <0.05 was regarded as statistically significant (* *p* < 0.05, ** *p* < 0.01).

## 4. Discussion

Although previous studies have demonstrated the anti-inflammatory properties of quercetin in various inflammatory responses and diseases, they have mainly focused on the priming step of inflammatory responses and canonical inflammasomes, particularly the NLRP3 inflammasome [29,30,31,32]. The regulatory role of quercetin in noncanonical inflammasome activation, however, remains unexplored. This study, therefore, aimed to investigate the anti-inflammatory effects of quercetin in caspase-11 noncanonical inflammasome-activated inflammatory responses in macrophages and acute lethal septic mice.

Selecting optimal doses of quercetin that show anti-inflammatory activity but no or minimal cytotoxicity was critical for this study. We first assessed the cytotoxicity profile of quercetin in macrophages and its protective effect against LPS-induced damage. Quercetin was found to be non-cytotoxic at doses up to 20 μM in J774A.1 cells and provided protection against LPS-mediated damage. This result is consistent with findings from other studies, where quercetin demonstrated low or minimal cytotoxicity at 12.5 and 25 μM in another type of macrophage, RAW264.7 cells [36]. As described earlier, quercetin is found in fruits, vegetables, and various plants, and people consume it more for preventive than therapeutic purposes. Therefore, for the in vitro study, macrophages were pretreated with quercetin before activating caspase-11 noncanonical inflammasome. LDH release is a cardinal feature resulting from the noncanonical inflammasome-activated pyroptosis [17,18,19,21,23]. The inhibitory effect of quercetin on pyroptosis increased in a dose-dependent manner up to 15 μM but decreased at 20 μM in J774A.1 cells. Based on this evidence, 10 and 15 μM doses of quercetin were selected for further experiments.

Quercetin effectively inhibited pyroptosis-induced cell death and LDH release and protected against pyroptosis-mediated damage in caspase-11 noncanonical inflammasome-activated J774A.1 cells. This strongly suggests that quercetin plays an in vitro anti-inflammatory role by protecting macrophages from pyroptosis-induced cell damage and death during caspase-11 noncanonical inflammasome-activated inflammatory responses. However, diclofenac exhibited no protective effect against pyroptosis in macrophages. This result aligns with previous findings that diclofenac, even at higher doses (50 μM) than those used in this study (15 μM) did not show a protective effect against pyroptosis in macrophages [17,18,19,21,23]. This result suggests that quercetin has a more potent inhibitory effect than diclofenac on pyroptosis in macrophages.

Another key feature of noncanonical inflammasome activation is the release of pro-inflammatory cytokines IL-1β and IL-18, which are specifically activated and released through GSDMD pores in response to inflammasome activation [8,9]. Quercetin inhibited the release of both IL-1β and IL-18 in a dose-dependent manner in caspase-11 noncanonical inflammasome-activated J774A.1 cells. Additionally, quercetin inhibited the release of IL-6 in these cells. However, unlike IL-1β and IL-18, IL-6 was released in Pam3CSK4-primed J774A.1 cells, indicating that IL-6 is a common inflammation marker in infection and inflammatory processes, and it is not released mainly through GSDMD pores during pyroptosis but secreted through the classical synthesis and secretory pathways (i.e., golgi apparatus). Notably, quercetin did not inhibit the release of TNF-a in caspase-11 noncanonical inflammasome-activated J774A.1 cells. We further investigated the inhibitory effect of quercetin on the gene expression of pro-inflammatory cytokines. Quercetin dose-dependently decreased the gene expression of IL-1β, IL-18, and IL-6 in caspase-11 noncanonical inflammasome-activated J774A.1 cells; however, it did not inhibit the gene expression of TNF-α. These findings indicate that quercetin inhibits the release of pro-inflammatory cytokines by suppressing pyroptosis in macrophages. However, the mechanism by which quercetin decreases the gene expression of these pro-inflammatory cytokines in caspase-11 noncanonical inflammasome-activated macrophages remains unclear. Some studies suggest that quercetin may decrease the gene expression of pro-inflammatory cytokines by inhibiting the nuclear factor-kappa B signaling pathway in macrophages [36,37]. Notably, diclofenac only inhibited the release and gene expression of IL-1β in caspase-11 noncanonical inflammasome-activated J774A.1 cells, with a lower efficiency than quercetin. This indicates that diclofenac, a cyclooxygenase inhibitor, plays an anti-inflammatory role through different mechanisms compared with quercetin during caspase-11 noncanonical inflammasome-activated inflammatory responses in macrophages.

NO production and increased gene expression of iNOS, an enzyme for catalyzing NO production, are key features of the priming step of inflammatory responses in macrophages [38,39,40]. Our previous studies have revealed that NO production and *iNOS* gene expression are also highly increased during caspase-11 noncanonical inflammasome-activated inflammatory responses in macrophages [17,18,19,21,23]. Therefore, we evaluated the inhibitory effect of quercetin on NO production and *iNOS* gene expression in caspase-11 noncanonical inflammasome-activated inflammatory responses in macrophages. We observed that quercetin inhibited NO production in both LPS-stimulated and caspase-11 noncanonical inflammasome-activated J774A.1 cells and decreased the expression of the *iNOS* gene in caspase-11 noncanonical inflammasome-activated J774A.1 cells. Previous studies have reported the inhibitory effect of quercetin on NO production and *iNOS* gene expression in LPS-primed macrophages [36,37], suggesting that quercetin inhibits these processes during both LPS-primed and caspase-11 noncanonical inflammasome-activated inflammatory responses in macrophages. Although diclofenac also inhibited NO production and *iNOS* gene expression in caspase-11 noncanonical inflammasome-activated J774A.1 cells, its effect was considerably lower than that of quercetin, suggesting that quercetin possesses superior anti-inflammatory properties by inhibiting the production and gene expression of inflammatory molecules and enzymes in macrophages.

Next, we investigated the molecular mechanism by which quercetin inhibits pyroptosis and the production of inflammatory molecules induced by caspase-11 noncanonical inflammasome activation in macrophages. The most critical process in caspase-11 noncanonical inflammasome activation is the autoproteolytic cleavage of the inactive pro-caspase-11 into its active form, followed by the release of active caspase-11 from the cells [41]. We evaluated the inhibitory effect of quercetin on the autoproteolytic cleavage of pro-caspase-11 and the release of caspase-11 in macrophages, finding that quercetin dose-dependently inhibited both the autoproteolytic cleavage of pro-caspase-11 and the release of active caspase-11 in caspase-11 noncanonical inflammasome-activated J774A.1 cells. The inactive pro-caspase-11 was not released from the cells, possibly because it was not cleaved to the active caspase-11. These results suggest that quercetin inhibits not only the priming step of inflammatory responses but also the triggering step by suppressing the autoproteolytic activation of caspase-11 noncanonical inflammasome in macrophages. Another critical step in caspase-11 noncanonical inflammasome activation is the proteolytic cleavage of full-length GSDMD into N-GSDMD, which produces GSDMD pores and results in GSDMD pore-mediated pyroptosis [8,9]. We assessed the inhibitory effect of quercetin on the proteolytic cleavage of GSDMD in macrophages and observed that quercetin completely inhibited the proteolytic cleavage of GSDMD and the generation of N-GSDMD in caspase-11 noncanonical inflammasome-activated J774A.1 cells. These results are consistent with the quercetin-mediated inhibition of pyroptosis in J774A.1 cells. These results also suggest that quercetin inhibits the production and release of pro-inflammatory cytokines via two mechanisms: (1) decreasing the gene expression of pro-inflammatory cytokines and (2) inhibiting GSDMD pore generation and GSDMD pore-mediated pyroptosis in macrophages. Activation of the caspase-11 noncanonical inflammasome is triggered by the direct recognition of cytosolic LPS by caspase-11 [8,9]. Therefore, we evaluated the inhibitory effect of quercetin on caspase-11-mediated direct recognition of LPS. Notably, quercetin did not inhibit the direct interaction between caspase-11 and LPS, indicating that quercetin-mediated inhibitory effect on caspase-11 noncanonical inflammasome-activated inflammatory responses is achieved by suppressing the proteolytic activation of caspase-11 and GSDMD rather than by interfering with the caspase-11-mediated direct recognition of cytosolic LPS in macrophages.

The in vivo anti-inflammatory effect of quercetin was evaluated using a mouse model of LPS-induced acute lethal sepsis, a well-established model for studying caspase-11 noncanonical inflammasome activation. In this model, LPS injection activates the caspase-11 noncanonical inflammasome in mice, leading to caspase-11 noncanonical inflammasome-activated inflammatory responses and the pathogenesis of acute lethal sepsis [5,6,7]. Quercetin significantly increased the survival rate of the mice and reduced serum levels of IL-1β and IL-18. A higher dose of quercetin (50 mg/kg) resulted in a better survival rate and a greater reduction in serum release of IL-18, while the lower dose of quercetin (25 mg/kg) was more effective at inhibiting serum IL-1β release. This discrepancy suggests that the two doses used in this study might not be optimal, highlighting the need for further research to identify and validate the optimal doses of quercetin for its pharmacological action. The body weights of the diseased mice in all groups remained consistent, and no significant abnormal behaviors were observed, indicating that quercetin was well tolerated at both doses throughout the experimental period and did not exhibit significant toxicity. However, the duration of the experiment (72 h) may not be long enough to fully assess the in vivo toxicity or adverse effects in the diseased mice. Long-term observation might be helpful in determining the in vivo toxicity of quercetin in acute lethal septic mice.

**Figure 5:**
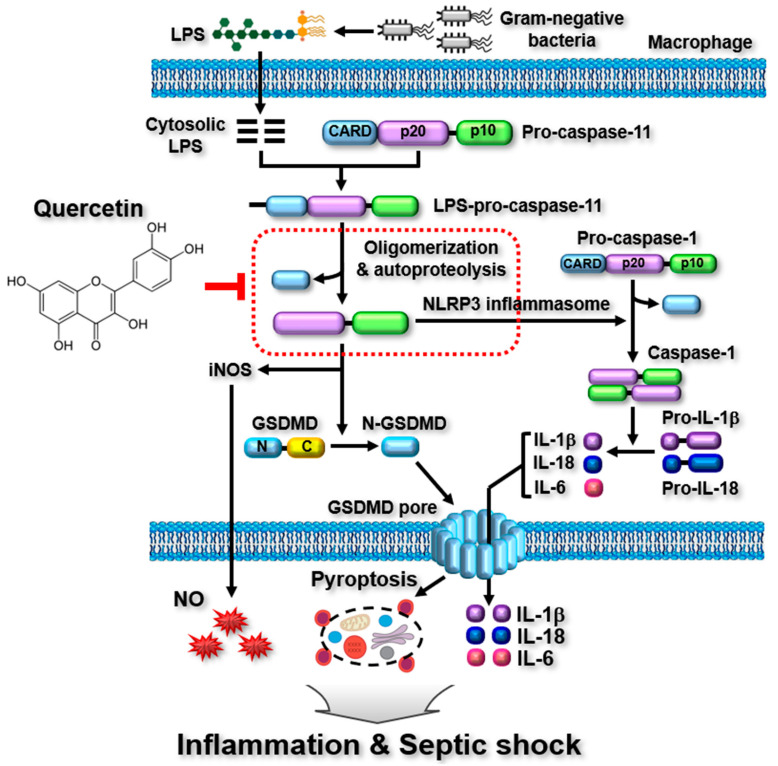
Graphical summary of quercetin-mediated anti-inflammatory action through targeting caspase-11 noncanonical inflammasome in macrophages. Pro-caspase-11 senses intracellular LPS through direct interaction, forming LPS-pro-caspase-11 complexes, which then undergo oligomerization to generate the caspase-11 non-canonical inflammasome. The caspase-11 noncanonnical inflammasome is activate by the autoproteolysis of caspase-11, leading to: (1) the proteolytic activation of GSDMD and GSDMD pore-induced pyroptosis, (2) iNOS-catalyzed NO production, and (3) the caspase-1-mediated proteolytic maturation and release of pro-inflammatory cytokines through the GSDMD pores in macrophages. Quercetin inhibits the autoproteolytic activation of the caspase-11 noncanonical inflammasome (red box), leading to the suppression of pyroptosis and the release of pro-inflammatory cytokines and NO in macrophages. Additionally, it ameliorates LPS-induced acute lethal sepsis in mice. Black arrows indicate the activation pathways.

This study demonstrated that quercetin alleviates the inflammatory responses activated by caspase-11 noncanonical inflammasome in macrophages and ameliorates acute lethal sepsis in mice, as shown in Figure 5. Although quercetin has been previously reported to play an anti-inflammatory role in macrophage-mediated inflammatory responses by inhibiting the priming step of inflammatory responses or NLRP3 canonical inflammasome activation [29,30,31,32], this study is the first to report its anti-inflammatory activity through a novel mechanism that inhibits the activation of caspase-11 noncanonical inflammasome in macrophages. These findings suggest that quercetin plays a multifaceted anti-inflammatory role, inhibiting not only the priming step of inflammatory responses but also the activation of both canonical and noncanonical inflammasomes in the triggering step of inflammatory responses in macrophages. Despite the strong evidence for quercetin-mediated anti-inflammatory action in this study, certain limitations need to be addressed. First, the optimal doses of quercetin for achieving the best in vitro and in vivo anti-inflammatory effects should be determined and validated. A lower dose of quercetin showed a better inhibitory effect on serum release of IL-1β in the diseased mice, indicating that a higher dose is not an optimal dose for the acute lethal septic mice used in this study. Second, this study demonstrated the inhibitory role of quercetin in caspase-11 noncanonical inflammasome in mouse macrophages and in a mouse model of acute lethal sepsis. Given that caspase-4 is the human homolog of mouse caspase-11, the inhibitory roles of quercetin in caspase-4 noncanonical inflammasome-activated inflammatory responses and diseases should be investigated in human monocytes such as THP-1 and U937 cells. Third, as the pharmacological action of quercetin was only evaluated in cells and a mouse disease model, translational studies of quercetin are required in human patients with caspase-4 noncanonical inflammasome-induced diseases. Finally, although diclofenac was used as a control drug in this study, comparative studies using quercetin and other anti-inflammatory drugs, such as steroids and NSAIDs, or caspase-11 inhibitors, such as wedelolactone [42], would provide further insights into the potential of quercetin as a natural medicine for inflammatory and infectious diseases.

In conclusion, we have demonstrated the anti-inflammatory role of quercetin through a novel molecular mechanism by targeting the caspase-11 noncanonical inflammasome in macrophages and in a mouse model of acute lethal sepsis. This study provides a scientific understanding of quercetin-mediated anti-inflammatory properties and the underlying novel molecular mechanisms, and it highlights the potential of quercetin-based anti-inflammatory therapeutics for the prevention and treatment of human inflammatory and infectious diseases.

## Figures and Tables

**Table 1:** The *p* values of the survival rate between groups (* *p* < 0.05).

Group	Vs.	Group	*p* Value
Vehicle		Quercetin (25 mg/kg)	0.031572 *
Vehicle		Quercetin (50 mg/kg)	0.013279 *
Quercetin (25 mg/kg)		Quercetin (50 mg/kg)	0.317311

**Table 2:** Primer sequences used for qPCR.

Target			Sequence (5’ to 3’)
Mouse	IL-1β	For	GTGAAATGCCACCTTTTGACAGTG
		Rev	CCTGCCTGAAGCTCTTGTTG
Mouse	IL-18	For	CAGCCTGTGTTCGAGGATATG
		Rev	TCACAGCCAGTCCTCTTACT
Mouse	TNF-α	For	TGCCTATGTCTCAGCCTCTTC
		Rev	GAGGCCATTTGGGAACTTCT
Mouse	IL-6	For	GACAAAGCCAGAGTCCTTCAGAGA
		Rev	CTAGGTTTGCCGAGTAGATCTC
Mouse	iNOS	For	GCCACCAACAATGGCAACAT
		Rev	TCGATGCACAACTGGGTGAA
Mouse	GAPDH	For	CAATGAATACGGCTACAGCAAC
		Rev	AGGGAGATGCTCAGTGTT

## Data Availability

Data are contained within the article.

## References

[1] Janeway C.A., Medzhitov R. (2002). Innate immune recognition. Annu. Rev. Immunol..

[2] Yi Y.S. (2020). Functional crosstalk between non-canonical caspase-11 and canonical NLRP3 inflammasomes during infection-mediated inflammation. Immunology.

[3] Zheng D., Liwinski T., Elinav E. (2020). Inflammasome activation and regulation: Toward a better understanding of complex mechanisms. Cell Discov..

[4] Xue Y., Enosi Tuipulotu D., Tan W.H., Kay C., Man S.M. (2019). Emerging Activators and Regulators of Inflammasomes and Pyroptosis. Trends Immunol..

[5] Kayagaki N., Wong M.T., Stowe I.B., Ramani S.R., Gonzalez L.C., Akashi-Takamura S., Miyake K., Zhang J., Lee W.P., Muszynski A. (2013). Noncanonical inflammasome activation by intracellular LPS independent of TLR4. Science.

[6] Hagar J.A., Powell D.A., Aachoui Y., Ernst R.K., Miao E.A. (2013). Cytoplasmic LPS activates caspase-11: Implications in TLR4-independent endotoxic shock. Science.

[7] Shi J., Zhao Y., Wang Y., Gao W., Ding J., Li P., Hu L., Shao F. (2014). Inflammatory caspases are innate immune receptors for intracellular LPS. Nature.

[8] Yi Y.S. (2017). Caspase-11 non-canonical inflammasome: A critical sensor of intracellular lipopolysaccharide in macrophage-mediated inflammatory responses. Immunology.

[9] Yi Y.S. (2018). Regulatory Roles of the Caspase-11 Non-Canonical Inflammasome in Inflammatory Diseases. Immune Netw..

[10] Li Y., Huang H., Liu B., Zhang Y., Pan X., Yu X.Y., Shen Z., Song Y.H. (2021). Inflammasomes as therapeutic targets in human diseases. Signal Transduct. Target. Ther..

[11] Bulte D., Rigamonti C., Romano A., Mortellaro A. (2023). Inflammasomes: Mechanisms of Action and Involvement in Human Diseases. Cells.

[12] Byun D.J., Lee J., Yu J.W., Hyun Y.M. (2023). NLRP3 Exacerbate NETosis-Associated Neuroinflammation in an LPS-Induced Inflamed Brain. Immune Netw..

[13] Chae B.J., Lee K.S., Hwang I., Yu J.W. (2023). Extracellular Acidification Augments NLRP3-Mediated Inflammasome Signaling in Macrophages. Immune Netw..

[14] Yi Y.S. (2024). MicroRNA-mediated epigenetic regulation of inflammasomes in inflammatory responses and immunopathologies. Semin. Cell Dev. Biol..

[15] Yi Y.S. (2022). Potential benefits of ginseng against COVID-19 by targeting inflammasomes. J. Ginseng Res..

[16] Ye J., Zeng B., Zhong M., Li H., Xu L., Shu J., Wang Y., Yang F., Zhong C., Ye X. (2021). Scutellarin inhibits caspase-11 activation and pyroptosis in macrophages via regulating PKA signaling. Acta Pharm. Sin. B.

[17] Cho H.J., Kim E., Yi Y.S. (2023). Korean Red Ginseng Saponins Play an Anti-Inflammatory Role by Targeting Caspase-11 Non-Canonical Inflammasome in Macrophages. Int. J. Mol. Sci..

[18] Cho H.J., Lee D.J., Yi Y.S. (2023). Anti-inflammatory activity of calmodulin-lysine N-methyltransferase through suppressing the caspase-11 non-canonical inflammasome. Immunobiology.

[19] Kim Y.B., Cho H.J., Yi Y.S. (2023). Anti-inflammatory role of Artemisia argyi methanol extract by targeting the caspase-11 non-canonical inflammasome in macrophages. J. Ethnopharmacol..

[20] Yi Y.S. (2023). Regulatory Roles of Flavonoids in Caspase-11 Non-Canonical Inflammasome-Mediated Inflammatory Responses and Diseases. Int. J. Mol. Sci..

[21] Joon Lee D., Yeol Lee S., Yi Y.S. (2024). Maclurin inhibits caspase-11 non-canonical inflammasome in macrophages and ameliorates acute lethal sepsis in mice. Int. Immunopharmacol..

[22] Yi Y.S. (2024). Roles of the Caspase-11 Non-Canonical Inflammasome in Rheumatic Diseases. Int. J. Mol. Sci..

[23] Min J.H., Cho H.J., Yi Y.S. (2022). A novel mechanism of Korean Red Ginseng-mediated anti-inflammatory action via targeting caspase-11 non-canonical inflammasome in macrophages. J. Ginseng Res..

[24] Yi Y.S. (2022). Dual roles of the caspase-11 non-canonical inflammasome in inflammatory bowel disease. Int. Immunopharmacol..

[25] Yi Y.S. (2022). Regulatory Roles of Caspase-11 Non-Canonical Inflammasome in Inflammatory Liver Diseases. Int. J. Mol. Sci..

[26] Singh P., Arif Y., Bajguz A., Hayat S. (2021). The role of quercetin in plants. Plant Physiol. Biochem..

[27] Batiha G.E., Beshbishy A.M., Ikram M., Mulla Z.S., El-Hack M.E.A., Taha A.E., Algammal A.M., Elewa Y.H.A. (2020). The Pharmacological Activity, Biochemical Properties, and Pharmacokinetics of the Major Natural Polyphenolic Flavonoid: Quercetin. Foods.

[28] Carrillo-Martinez E.J., Flores-Hernandez F.Y., Salazar-Montes A.M., Nario-Chaidez H.F., Hernandez-Ortega L.D. (2024). Quercetin, a Flavonoid with Great Pharmacological Capacity. Molecules.

[29] Li Y., Yao J., Han C., Yang J., Chaudhry M.T., Wang S., Liu H., Yin Y. (2016). Quercetin, Inflammation and Immunity. Nutrients.

[30] Shen P., Lin W., Deng X., Ba X., Han L., Chen Z., Qin K., Huang Y., Tu S. (2021). Potential Implications of Quercetin in Autoimmune Diseases. Front. Immunol..

[31] Blevins H.M., Xu Y., Biby S., Zhang S. (2022). The NLRP3 Inflammasome Pathway: A Review of Mechanisms and Inhibitors for the Treatment of Inflammatory Diseases. Front. Aging Neurosci..

[32] Aghababaei F., Hadidi M. (2023). Recent Advances in Potential Health Benefits of Quercetin. Pharmaceuticals.

[33] Mosmann T. (1983). Rapid colorimetric assay for cellular growth and survival: Application to proliferation and cytotoxicity assays. J. Immunol. Methods.

[34] Guevara I., Iwanejko J., Dembinska-Kiec A., Pankiewicz J., Wanat A., Anna P., Golabek I., Bartus S., Malczewska-Malec M., Szczudlik A. (1998). Determination of nitrite/nitrate in human biological material by the simple Griess reaction. Clin. Chim. Acta.

[35] Koontz L. (2014). TCA precipitation. Methods Enzymol..

[36] Yang W.S., Jeong D., Yi Y.S., Lee B.H., Kim T.W., Htwe K.M., Kim Y.D., Yoon K.D., Hong S., Lee W.S. (2014). Myrsine seguinii ethanolic extract and its active component quercetin inhibit macrophage activation and peritonitis induced by LPS by targeting to Syk/Src/IRAK-1. J. Ethnopharmacol..

[37] Endale M., Park S.C., Kim S., Kim S.H., Yang Y., Cho J.Y., Rhee M.H. (2013). Quercetin disrupts tyrosine-phosphorylated phosphatidylinositol 3-kinase and myeloid differentiation factor-88 association, and inhibits MAPK/AP-1 and IKK/NF-kappaB-induced inflammatory mediators production in RAW 264.7 cells. Immunobiology.

[38] Yi Y.S., Kim H.G., Kim J.H., Yang W.S., Kim E., Jeong D., Park J.G., Aziz N., Kim S., Parameswaran N. (2021). Syk-MyD88 Axis Is a Critical Determinant of Inflammatory-Response in Activated Macrophages. Front. Immunol..

[39] Yi Y.S., Son Y.J., Ryou C., Sung G.H., Kim J.H., Cho J.Y. (2014). Functional roles of Syk in macrophage-mediated inflammatory responses. Mediat. Inflamm..

[40] Kwon K.W., Jang W.Y., Kim J.W., Noh J.K., Yi D.K., Cho J.Y. (2023). Anti-Inflammatory Effect of Meriania hexamera Sprague by Targeting Syk Kinase in NF-kappaB Signaling. Plants.

[41] Lee B.L., Stowe I.B., Gupta A., Kornfeld O.S., Roose-Girma M., Anderson K., Warming S., Zhang J., Lee W.P., Kayagaki N. (2018). Caspase-11 auto-proteolysis is crucial for noncanonical inflammasome activation. J. Exp. Med..

[42] Kobori M., Yang Z., Gong D., Heissmeyer V., Zhu H., Jung Y.K., Gakidis M.A., Rao A., Sekine T., Ikegami F. (2004). Wedelolactone suppresses LPS-induced caspase-11 expression by directly inhibiting the IKK complex. Cell Death Differ..

